# Cracking the Code: A Case Report on Low-Renin Hypertension

**DOI:** 10.7759/cureus.65335

**Published:** 2024-07-25

**Authors:** Fawwad A Ansari, Bilal Hamid, Inemesit Akpan, Ghida Akhdar, Muhammad Umer Riaz Gondal

**Affiliations:** 1 Internal Medicine, Piedmont Athens Regional Medical Center, Athens, USA; 2 Shifa Clinical Research Center, Shifa International Hospital Islamabad, Islamabad, PAK; 3 Internal Medicine, Reading Hospital, West Reading, USA

**Keywords:** low-renin hypertension, spironolactone, secondary hypertension, resistant hypertension, primary hyperaldosteronism

## Abstract

Low-renin hypertension (LRH) is characterized by hypertension accompanied by low serum renin levels. LRH is a spectrum, including low-renin essential hypertension (LREH), primary hyperaldosteronism, and several acquired or familial secondary forms. Here, we present a case of LRH. A 57-year-old female with resistant hypertension on multiple antihypertensive medications presented for blood pressure management. Workup for secondary causes of hypertension revealed low renin levels with normal aldosterone. The patient was initiated on spironolactone and responded quickly with normal blood pressure on a follow-up visit. LRH is an under-recognized etiology for uncontrolled hypertension. It can be secondary to several different causes. Although treatment of LREH is essentially the same as regular patients, these patients tend to respond well to sodium-volume-depleting diuretics, mineralocorticoid receptor blockers, and epithelial sodium channels (ENaC) blockers.

## Introduction

Low-renin hypertension (LRH) is one of the subsets of hypertension marked by decreased levels of an enzyme called renin. Renin is made in the juxta glomerular cells of the renal interstitium. It converts the precursor globulin produced by the liver called angiotensinogen, to angiotensin I, which is then converted to angiotensin II by the angiotensin-converting enzyme found in the lungs. The renin-angiotensin-aldosterone system is responsible for maintaining fluid balance and sodium and potassium homeostasis in the body. The prevalence of LRH is 20-30% in patients with hypertension [1]. However, the prevalence is higher in hypertensive patients of African descent [1]. LRH entails a broad spectrum of disorders, including the most prevalent low-renin essential hypertension (LREH), primary hyperaldosteronism, and several acquired or familial secondary forms. Thus, it is necessary to assess the renin status in managing arterial hypertension [1]. Dependable prediction of the response to treatment can be made from parameters that include plasma renin activity, age, sex, and race of the patients. Since people with resistant hypertension can have underlying low renin levels as a cause, they must be screened upon presentation so that an appropriate diagnosis can be made, which can guide treatment. Here, we present a middle-aged female with resistant hypertension who was ultimately diagnosed with LRH. 

## Case presentation

A 57-year-old female with a history of chronic back pain and long-standing hypertension presented to the resident clinic to establish care. She reported poorly controlled hypertension for over a decade despite multiple work-ups and anti-hypertensive regimen changes. Her home blood pressure readings were consistently around 190/100 mmHg, even though she was taking four anti-hypertensive medications: amlodipine 10 mg daily, hydralazine 25 mg twice daily, atenolol 100 mg daily, and fixed-dose combination of losartan-hydrochlorothiazide 100/25 mg daily. She confirmed adherence to her medication regimen. Her family history was significant for hypertension in two daughters, her mother, a grandparent, and several aunts, some of whom were diagnosed in their early 20s. Regarding her lifestyle, she reported significantly reducing salt intake and fried foods. She denied cigarette use, alcohol, or substance use.

At the time of the clinic visit, her blood pressure was elevated at 172/96 mmHg, her heart rate was 92 beats per minute, and her respiratory rate was 18 cycles per minute. Physical examination, including cardiac examination, was unremarkable. She denied any headache, focal weakness or numbness, asymmetry of face or slurring of speech, chest pain, palpitations, shortness of breath, orthopnea, paroxysmal nocturnal dyspnea, and swelling of the lower limbs.

Lifestyle modifications were reinforced, and the dose of hydralazine was increased from 25 mg twice daily to 50 mg twice daily. The patient was advised to continue the rest of her medications without any changes. A work-up was ordered for evaluation for secondary causes of hypertension. On a follow-up appointment, her blood pressure was still very uncontrolled, with no significant change despite lifestyle changes and medication adherence.

Laboratory investigation (Table 1) revealed potassium of 3.8 mmol/L, normal kidney function with creatinine of 0.65 mg/dL, and thyroid-stimulating hormone (TSH) of 1.751 uIU/mL. The patient had suppressed renin of 0.09 ng/mL/h and aldosterone levels of 6 ng/dL, with an aldosterone/renin ratio of 66.7.

**Table 1:** Laboratory workup for the patient mmol/L: millimoles per liter, mg/dL: milligrams per deciliter, uIU/mL: micro–International Unit per milliliter, ng/mL/h: nanogram per milliliter per hour, ng/dL: nanograms per deciliter

Variable	Labs	Reference range
Potassium (mmol/L)	3.8	3.3-5.1
Creatinine (mg/dL)	065	0.4-1.00
TSH (uIU/mL)	1.751	0.340-0.500
Plasma renin activity (ng/mL/h)	0.09	0.25-5.82
Serum aldosterone (ng/dL)	6	<28
Aldosterone/renin ratio	66.7	0.9-28.9

Primary hyperaldosteronism was ruled out as plasma aldosterone levels were <10 ng/dL despite of elevated aldosterone/renin ratio. Due to suspicion of LRH, the patient was initiated on spironolactone 25 mg daily and was advised to discontinue atenolol 100 mg daily and losartan-hydrochlorothiazide 100-25 mg daily.

On a follow-up visit two weeks later, her blood pressure had improved to 139/84 mmHg, and she endorsed feeling much better with improved blood pressure reading at home. Although she was advised to follow up with endocrinology, she was lost to follow-up.

## Discussion

LRH has a high prevalence in the hypertensive population; however, it is underrecognized due to inadequate screening. It includes a wide range of disorders, including monogenic, secondary, and essential forms, the most common of which are LREH and primary aldosteronism. Based on a detailed family history and evaluation of serum aldosterone and potassium levels, a differential diagnosis can be made for which appropriate targeted therapy can be initiated [1].

**Figure 1:**
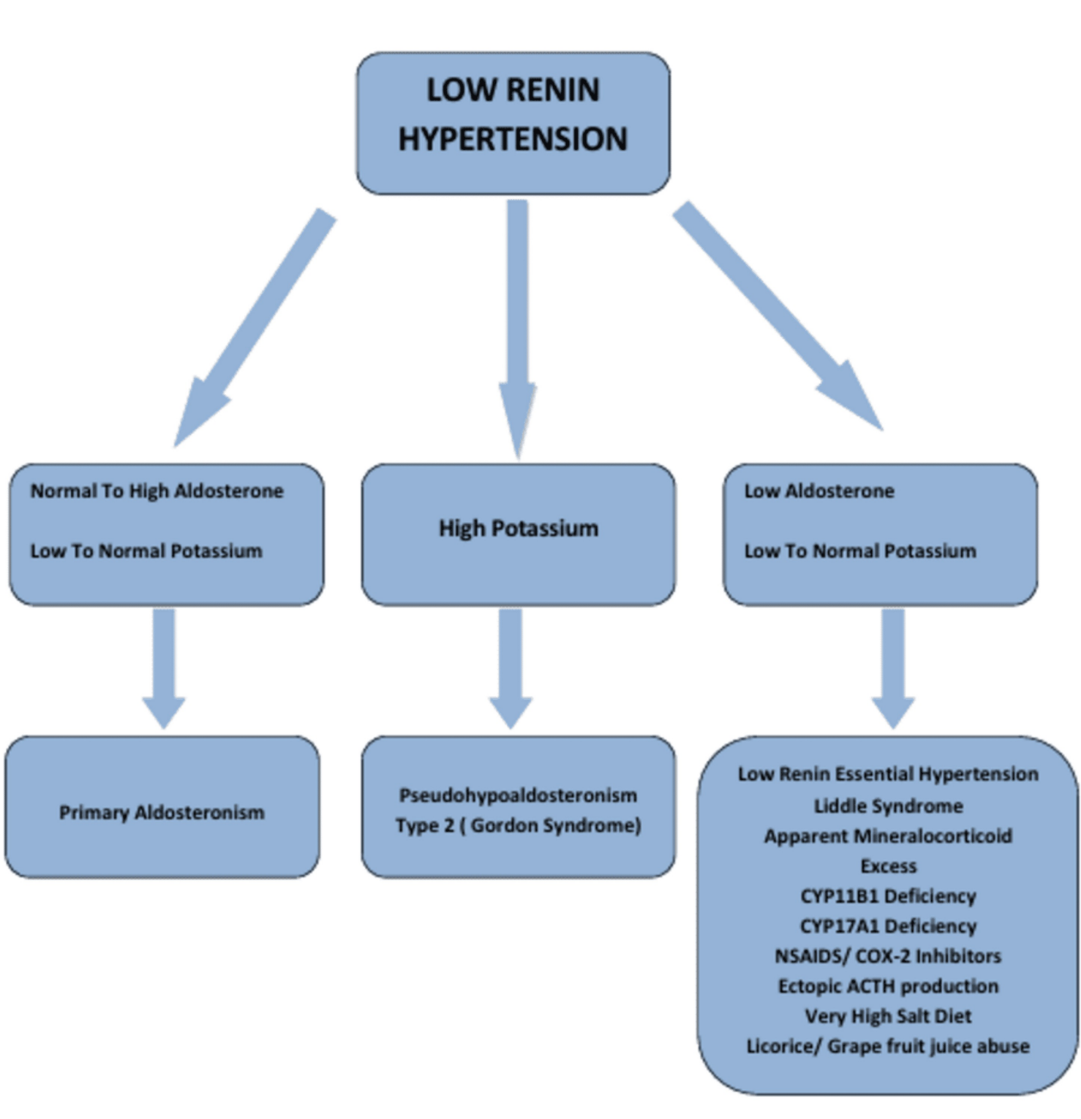
Differential diagnosis of low-renin hypertension based on the plasma renin activity, serum aldosterone, and potassium levels Reference: Monticone S, Losano I, Tetti M, Buffolo F, Veglio F, Mulatero P: Diagnostic approach to low‐renin hypertension. Clinical Endocrinology. 2018, 89:385-96.

Patients with hypertension can be divided into low-, normal-, or high-renin hypertension based on their serum renin levels. LRH presents with decreased levels of renin in the presence of low, normal, or high levels of plasma aldosterone [2]. When evaluating patients suspected of LRH, measuring plasma aldosterone and plasma potassium is fundamental to narrowing down the differential diagnosis. Among the many causes of LRH include primary aldosteronism (sporadic/familial), pseudo-hypoaldosteronism type 2 (Gordon syndrome), Liddle syndrome, apparent mineralocorticoid excess, CYP11B1 deficiency, CYP17A1 deficiency, mineralocorticoid receptor activating mutation, glucocorticoid resistance, licorice/grapefruit juice use, nonsteroidal anti-inflammatory drugs (NSAIDs), COX-2 inhibitors, heparin, ectopic adrenocorticotropic hormone (ACTH) production, deoxycorticosterone-producing tumors, very-high-salt diet, and LREH (Figure 1). Primary aldosteronism is suspected when decreased plasma renin activity (PRA) and increased aldosterone levels occur. After confirmation, further testing, including genetic testing, can be performed. 

The plasma aldosterone/plasma renin activity ratios (ARR) may sometimes be mildly elevated in LREH (>30 but <100) [3]. It is unclear whether there is a true mineralocorticoid excess in this group [3]. It has been argued that one of the pathophysiological mechanisms in LREH is increased renal salt absorption attributed to the activated ENaC complex, with the consequent increase in the volume and suppression of plasma renin activity. Patients with LREH typically tend to have normal aldosterone levels with suppressed renin levels. This is in contrast to Liddle’s syndrome, where plasma renin and aldosterone activity are markedly suppressed.

When dealing with ARR, it is important to be mindful that it can be affected by several factors, including dietary salt intake, medications, and posture during sample draw. Our patient was on losartan, which may cause falsely low ARR with high plasma renin activity [4]. Despite being on the medication, the patient had suppressed renin activity with an elevated ARR, indicating an underlying pathology.

LRH usually responds well to sodium volume-depleting natriuretic drugs, such as thiazide diuretics. Meanwhile, medium- and high-renin hypertension responds well to other antihypertensive medications, like angiotensin-converting enzyme inhibitors, angiotensin receptor blockers, and β-blockers, all of which antagonize plasma renin activity [5]. Treatment of patients with low renin essential hypertension is similar to that of patients with essential hypertension; however, since it has been found that mineralocorticoid excess plays a role in this condition, the addition of mineralocorticoid receptor antagonists such as spironolactone and eplerenone are often effective. Patients with resistant hypertension may be very sensitive to low-dose mineralocorticoid receptor antagonists and provide significant blood pressure reductions, as approximately 60-70% of resistant patients are generally found to have low renin. In patients with LRH, further addition of amiloride/hydrochlorothiazide has shown a better response than other diuretic combinations that did not include ENaC channel blockers [6]. Our presentation looks at a patient with uncontrolled hypertension resistant to the antihypertensives the patient was already taking. Upon further evaluation, it was found that she had decreased serum renin levels with normal aldosterone levels. The serum aldosterone to renin ratio was found to be elevated. Diagnosis of LRH (likely LREH) was presumptively made, and the patient was started on spironolactone. Additional information and tests were required further to narrow down the etiology for LRH; however, the patient was lost to follow-up. It is, therefore, of utmost importance to recognize this classification and know about the different causes of LRH to initiate the appropriate workup and treatment plan.

## Conclusions

LRH is an under-recognized etiology for uncontrolled hypertension. It must be considered in the differentials, and a workup should be done to determine the cause of low renin. Although treatment of LREH is essentially the same as that of regular patients, these patients tend to respond well to sodium-volume-depleting diuretics, mineralocorticoid receptor blockers, and ENaC blockers.
